# A pathogenic role for the integrin CD103 in experimental allergic airways disease

**DOI:** 10.14814/phy2.13021

**Published:** 2016-11-15

**Authors:** Vanessa S. Fear, Siew Ping Lai, Graeme R. Zosky, Kara L. Perks, Shelley Gorman, Fabian Blank, Christophe von Garnier, Philip A. Stumbles, Deborah H. Strickland

**Affiliations:** ^1^ Telethon Kids Institute Perth Australia; ^2^ Department of Clinical Research University of Bern Bern Switzerland; ^3^ School of Veterinary and Life Sciences Murdoch University Perth Australia; ^4^ School of Paediatrics and Child Health University of Western Australia Perth Australia

**Keywords:** Allergy, asthma, CD103, CD4 T cell, dendritic cell

## Abstract

The integrin CD103 is the *α*_E_ chain of integrin *α*_E_ *β*_7_ that is important in the maintenance of intraepithelial lymphocytes and recruitment of T cells and dendritic cells (DC) to mucosal surfaces. The role of CD103 in intestinal immune homeostasis has been well described, however, its role in allergic airway inflammation is less well understood. In this study, we used an ovalbumin (OVA)‐induced, CD103‐knockout (KO) BALB/c mouse model of experimental allergic airways disease (EAAD) to investigate the role of CD103 in disease expression, CD4^+^ T‐cell activation and DC activation and function in airways and lymph nodes. We found reduced airways hyper‐responsiveness and eosinophil recruitment to airways after aerosol challenge of CD103 KO compared to wild‐type (WT) mice, although CD103 KO mice showed enhanced serum OVA‐specific IgE levels. Following aerosol challenge, total numbers of effector and regulatory CD4^+^ T‐cell subsets were significantly increased in the airways of WT but not CD103 KO mice, as well as a lack of DC recruitment into the airways in the absence of CD103. While total airway DC numbers, and their in vivo allergen capture activity, were essentially normal in steady‐state CD103 KO mice, migration of allergen‐laden airway DC to draining lymph nodes was disrupted in the absence of CD103 at 24 h after aerosol challenge. These data support a role for CD103 in the pathogenesis of EAAD in BALB/c mice through local control of CD4^+^ T cell and DC subset recruitment to, and migration from, the airway mucosa during induction of allergic inflammation.

## Introduction

The integrin CD103 is the *α* chain of integrin *α*_E_ *β*_7_, an adhesion molecule that mediates cell binding primarily to the epithelial transmembrane glycoprotein E‐cadherin (Agace et al. 2000). CD103 expression has been well characterized on T cells in the intestinal tract of mice, where the majority (>90%) of intraepithelial T cells and a large proportion (40–50%) of mucosal T cells express CD103, compared to a small proportion (<15%) in the blood or spleen (Agace et al. 2000). In humans, CD103 is also preferentially expressed on CD4^+^ and CD8^+^ T cells in gut, where up to 80% of T cells can be CD103^+^ compared to less than 5% of T cells in peripheral blood (Cerf‐Bensussan et al. 1987). The CD103 molecule has been implicated in the retention of T cells in the gut epithelium and also in the skin (El‐Asady et al. 2005; Suffia et al. 2005), although additional studies have shown that CD103 is not essential for CD8^+^ T‐cell accumulation in the intestine, leading others to suggest that alternative ligands for CD103 may exist (Lefrançois et al. 1999; Strauch et al. 2001).

Beyond the T‐cell compartment, CD103 is reciprocally expressed with CD11b on subsets of dendritic cells (DC) in most lymphoid and nonlymphoid tissues of mice, including the intestinal mucosa and mesenteric lymph nodes where CD103^+^ DC are proposed to play a role in the regulation of intestinal inflammation via interactions with CD4^+^ regulatory T cells (Annacker et al. 2005; Ginhoux et al. 2009). In the lung, respiratory tract DC (RTDC) can be broadly classified into two major subsets based on the reciprocal expression of CD11b and CD103: CD11b^hi^ (CD103^‐^) RTDC express higher levels and a greater range of chemokines than CD103^+^ (CD11b^lo^) RTDC, while CD103^+^ RTDC express higher levels of Langerin and tight junction proteins and are thought to extend dendrites through epithelial tight junctions and into the airway lumen for antigen sampling (Sung et al. 2006; Beaty et al. 2007), (Hammad and Lambrecht 2008). Additionally, CD103^+^ DC appear to be specialized for cross‐presentation of inhaled proteins and viral antigens to CD8^+^ T cells, and have been shown to produce high levels of the Th1‐promoting cytokine IL‐12 (Sung et al. 2006; Helft et al. 2012; Nakano et al. 2012). In contrast, we have shown that CD11b^hi^ airway mucosal RTDC appear to preferentially capture inhaled proteins under steady‐state conditions for transport to airway draining lymph nodes (von Garnier et al. 2007; Wikstrom and Stumbles 2007), with others proposing that this subset of DC are specialized for presentation of inhaled antigens to CD4^+^ T cells in lymph nodes in the steady‐state (del Rio et al. 2007). However, we have also shown that CD103^+^ (CD11b^lo^) airway mucosal RTDC dramatically increase their capacity for inhaled antigen capture during allergic airways inflammation and promote allergen‐specific CD4^+^ T‐cell proliferation in airway draining lymph nodes (von Garnier et al. 2007).

To date, however, there is little information on the role of CD103‐expressing cells in the pathogenesis of allergic airways inflammation that underlies allergic asthma. The hallmarks of allergic asthma include airways hyper‐responsiveness (AHR) to inhaled allergen, with concomitant cellular infiltration of eosinophils, B cells, Th2 effector cells and RTDC, elevated allergen‐specific IgE and IgG1 antibody production and increased production of the pathogenic Th2‐cytokines IL‐4, IL‐5 and IL‐13 (Edelson et al. 2010). A number of studies have highlighted the central roles of both RTDC and CD4^+^ T cells in the pathogenesis of the disease, with RTDC required for inhaled allergen capture at the airway mucosa and transport to airway draining lymph node (ADLN) for activation of allergen‐specific CD4^+^ Th2 cells that mediate many of the clinical characteristics of the disease including IgE production, AHR and airway eosinophil recruitment (Holt et al. 2008).

In this study, we have examined the role of CD103 in the development of experimental allergic airways disease (EAAD) in a BALB/c mouse model of OVA sensitization and airways challenge. Although CD103 was not required for systemic priming of allergen‐specific IgE, CD103 expression was required for the local expression of the clinical signs of allergic airways inflammation, including CD4^+^ T‐cell influx into the airway mucosa, induction of AHR and airways eosinophilia. Importantly, we show that this was due to alterations in the capacity of airway mucosal RTDC to capture and transport inhaled allergen to draining lymph nodes, and for airway recruitment (but not lymph node priming) of effector CD4^+^ T‐cell subsets.

## Materials and Methods

### BALB/c mouse model of EAAD

Eight‐week old SPF female BALB/c mice were from the Animal Resource Centre, Perth, WA, Australia. BALB/c CD103^‐/‐^ (C. 129S2‐Itgae^tm1Cmp^/J) mice were purchased from Jackson Laboratories (Bar Harbor, ME) and bred at the Telethon Kids Institute. To induce EAAD, mice were sensitized by intraperitoneal (i.p.) injection of 20 *μ*g ovalbumin (OVA) in 200 *μ*L AlOH_3_ on days 0 and 14, then received 1% OVA aerosols for one or three consecutive days, or 50 *μ*g OVA in 50 *μ*L LPS‐free saline intranasally (i.n.) starting on day 21 as described (von Garnier et al. 2007). Control mice were sensitized to OVA as above and challenged with LPS‐free saline delivered as aerosol or i.n. All animal experiments were approved by the Telethon Kids Institute Animal Ethics Committee (AEC #240) operating under the National Health and Medical Research Council of Australia guidelines.

### BALF and serum collection

A duration of 24 h after the final aerosol, broncheoalveolar lavage fluid (BALF) was collected by flushing 1 ml of PBS into the lung as described (Burchell et al. 2009). Cells were cytocentrifuged onto glass slides and stained with DIFF‐Quik Stain (Lab Aids, Narrabeen, NSW, Australia). Sera was collected 24 h after the final aerosol by cardiac heart puncture and stored at −20°C.

### Assessment of AHR

A duration of 24 h after aerosol challenge, a modified low‐frequency forced oscillation technique (FOT) was used to measure change in respiratory input impedance (Zrs) in response to increasing doses of methacholine (0.1–30 mg/mL) as described previously (Burchell et al. 2009). The constant phase model was used to partition Zrs into components representing the conducting airway (airways resistance).

### Trachea and lymph node single cell preparations

Trachea and airway draining lymph node (ADLN) were prepared as single cell suspensions as described (von Garnier et al. 2005). Briefly, pooled (5 mice/group) trachea or ADLN were collagenase digested (Type IV; 1.5 mg/mL; Worthington Biochemical, Lakewood, NJ) and DNAse (0.1 mg/mL; Sigma Aldrich, Sydney, NSW, Australia), then washed in glucose potassium sodium buffer (GKN) as previously described (von Garnier et al. 2005).

### Flow cytometry

Cells were FcR blocked (2.4G2; BD Biosciences) prior to adding fluorochrome‐labeled monoclonal antibodies (mAbs) to I‐A/I‐E (2G9); CD11c (N418), CD11b (M1/70), CD8a (53–6.7), or CD103bio (M290) to identify DC, and CD3, CD4, CD62L and CD44 or CD3, CD4, CD25 (BD Biosciences, San Jose, CA) together with FoxP3 intracellular staining kit (eBiosciences, San Diego, CA) to identify CD4^+^ T‐cell subsets. All mAbs were used as direct conjugates to FITC, PE, PE‐Cy7, APC, APC‐Cy7, or biotin and streptavidin‐PE‐Cy5 (BD Biosciences). Samples were analyzed using an LSRII flow cytometer (BD Biosciences) and FlowJo software (FlowJo, Ashland, OR, USA)

### Assessment of inhaled antigen uptake in vivo

Mice were lightly anesthetized with gaseous isofluorane and then received 20 *μ*g OVA‐conjugated Alexa Fluor 647 (OVA‐A647; Molecular Probes; Eugene, OR, USA) in 50 *μ*L endotoxin‐free saline delivered i.n. on d21 following sensitization (Wikstrom and Stumbles 2007). Mice were killed at indicated time points after OVA‐A647 administration by sodium pentobarbital overdose, and trachea and ADLN tissue were harvested and analyzed by flow cytometry.

### Statistical analysis

Data were analyzed by unpaired, one‐ or two‐tailed Mann–Whitney *U‐*test using Prism 6 software (GraphPad, San Diego, CA).

## Results

### CD103 is required for the development of local airways hyper‐responsiveness and eosinophilia, but not systemic antigen‐specific IgE production, during induction of EAAD in BALB/c mice

**Figure 1:**
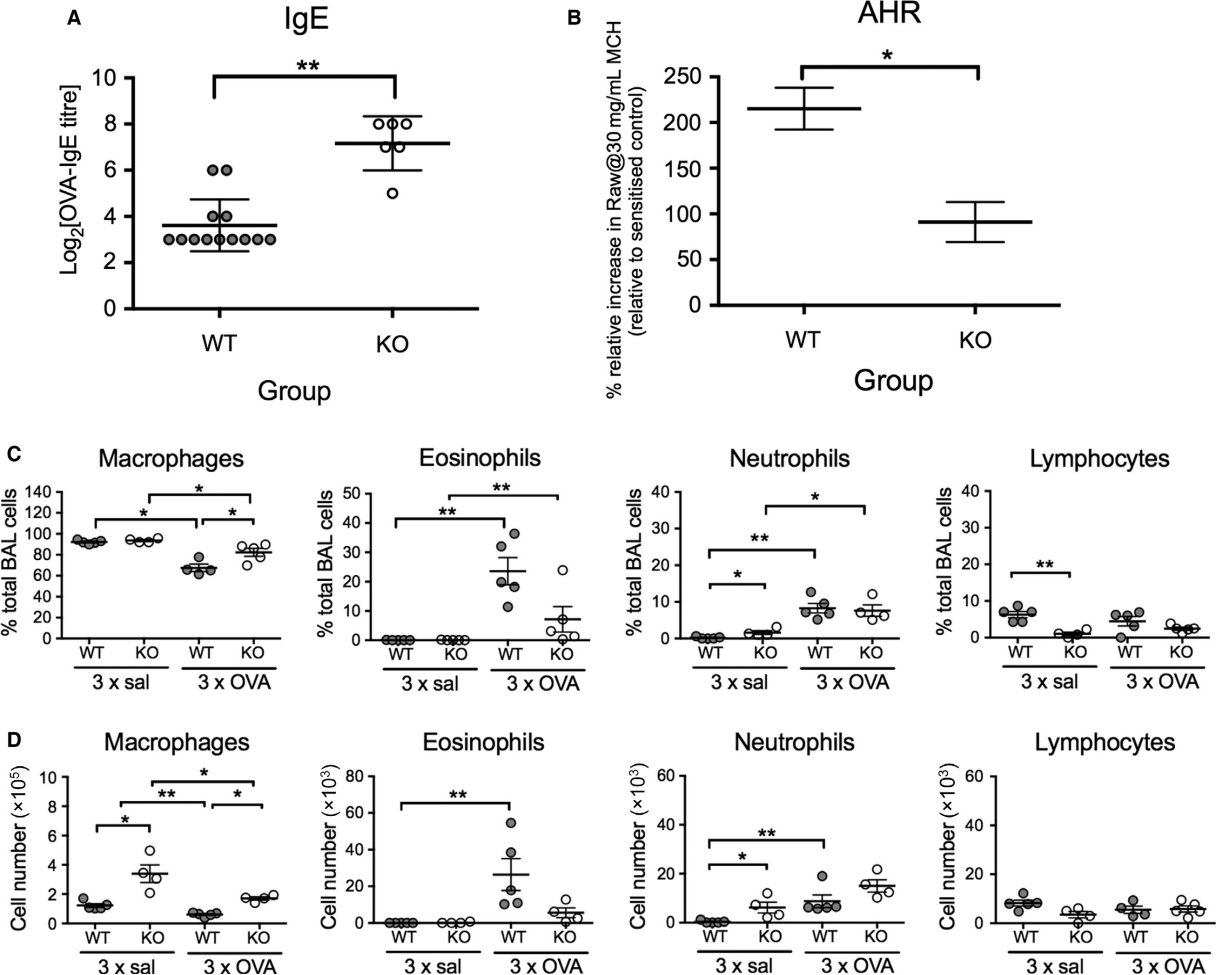
Clinical parameters of OVA‐induced experimental allergic airways disease in CD103^−/−^ and WT BALB/c mice. Adult BALB/c WT and CD103 KO mice were sensitized with OVA in aluminum hydroxide i.p. on days 0 and day 14, then challenged with either OVA or saline aerosols on days 21–23. (A) Sera were collected 24 h after last aerosol and OVA‐specific IgE determined by passive cutaneous anaphylaxis (PCA) as described in Materials and Methods (2 independent experiments, *n* > 6 mice/group/experiment). (B) Mice received a single OVA or saline aerosol challenge on day 21, 24 h prior to methacholine challenge (MCh) on day 22 and measurement of airways hyper‐responsiveness (AHR) by forced oscillation technique as described in Materials and Methods. Data indicate airways resistance, in response to 30 mg/ml MCh, in WT and CD103 KO mice relative to their respective OVA‐sensitized control. (Two independent experiments, *n* = 8 to 12 mice/group). (C) and (D) BALF was collected 24 h after final aerosol and the percentages (C) and total numbers (D) of macrophages, eosinophils, neutrophils, and lymphocytes determined by differential staining and counting as described in Materials and Methods (*n* = 5 mice/group; data are means ^+/−^ SEM). *P <* *0.05, **P <* *0.01 by two‐tailed Mann–Whitney *U*‐test. AHR, airways hyper‐responsiveness; BLF, Broncheoalveolar lavage fluid; WT, wild type; KO, knockout; OVA, ovalbumin.

To examine the potential role of CD103 in the development of the hallmark clinical features of allergic airways inflammation, we used a well‐established model of OVA‐induced EAAD in which adult BALB/c wild‐type (WT) or CD103 knock‐out (KO) mice were sensitized i.p. with OVA in AlOH_3_ (OVA‐Alum) on days 0 and 14, then OVA aerosol‐challenged with OVA on days 21, 22, and 23. We then examined the mice for development of circulating OVA‐specific IgE, airways hyper‐responsiveness (AHR), and BALF inflammatory cells 24 h later (Fig. 1). Notably, OVA‐sensitized and aerosol‐challenged KO mice developed significantly elevated levels of circulating OVA‐specific IgE (Fig. 1A; *P *<* *0.01), but significantly lower levels AHR in response to inhaled methacholine (Fig. 1B; *P *<* *0.05), when compared to WT mice. Nonsensitized and unchallenged control KO mice demonstrated a trend for increased baseline AHR to methacholine challenge compared to WT control mice. Unlike WT mice, sensitization and aerosol challenge of KO mice did not further increase AHR relative to baseline nonsensitized and unchallenged control levels (data not shown). Next, we examined the development of BALF cellular inflammation in response to allergen challenge by comparing the percentages (Fig. 1C) and total numbers (Fig. 1D) of macrophages, eosinophils, neutrophils, and lymphocytes in BALF of OVA‐sensitized and challenged WT and KO mice as compared to OVA‐sensitized and saline‐challenged controls. In saline‐challenged mice, there were no significant differences in the percentages of macrophages and eosinophils in BALF between WT and KO mice, however, KO mice showed a slightly increased percentage of neutrophils (*P *<* *0.05), and decreased percentage of lymphocytes (*P *<* *0.01), when compared to WT mice (Fig. 1C). Following OVA‐challenge, both WT and KO mice developed increased percentages of eosinophils (*P *<* *0.01) and neutrophils (*P *<* *0.01 and 0.05, respectively) and decreased percentages of macrophages (*P *<* *0.05) compared to saline controls, with the percentages of macrophages significantly lower in WT compared to KO OVA‐challenged mice (Fig. 1C). For total cell numbers, saline‐challenged KO mice showed significantly increased numbers of macrophages (*P *<* *0.05) and neutrophils (*P *<* *0.05) compared to WT mice, with no differences in eosinophil or lymphocyte numbers between saline‐challenged WT and KO mice (Fig. 1D). Following OVA‐challenge, WT mice developed significantly elevated numbers of eosinophils (*P *<* *0.01) and neutrophils (*P *<* *0.01) compared to saline‐challenge controls, whereas KO mice failed to develop elevated levels of either of these cell types compared to their saline‐challenged counterparts (Fig. 1D). Macrophage numbers were elevated in OVA‐challenged KO compared to WT mice (*P *<* *0.05), but decreased compared to saline‐challenged controls in both WT (*P *<* *0.01) and KO (*P *<* *0.05) mice, while no differences in lymphocyte numbers were observed for any group (Fig. 1D).

In summary, these data indicate that CD103 was required for the development of local, airway‐specific clinical signs of allergic airways inflammation in adult BALB/c mice following aeroallergen challenge, including AHR and BALF eosinophil responses. However, absence of CD103 did not impact on systemic allergen sensitization, as evidenced by a robust, and even enhanced, systemic antigen‐specific IgE response in CD103 KO mice.

### CD103 is required for effector and regulatory CD4^+^ T‐cell recruitment to the airway mucosa following antigen‐sensitization and aerosol challenge

**Figure 2:**
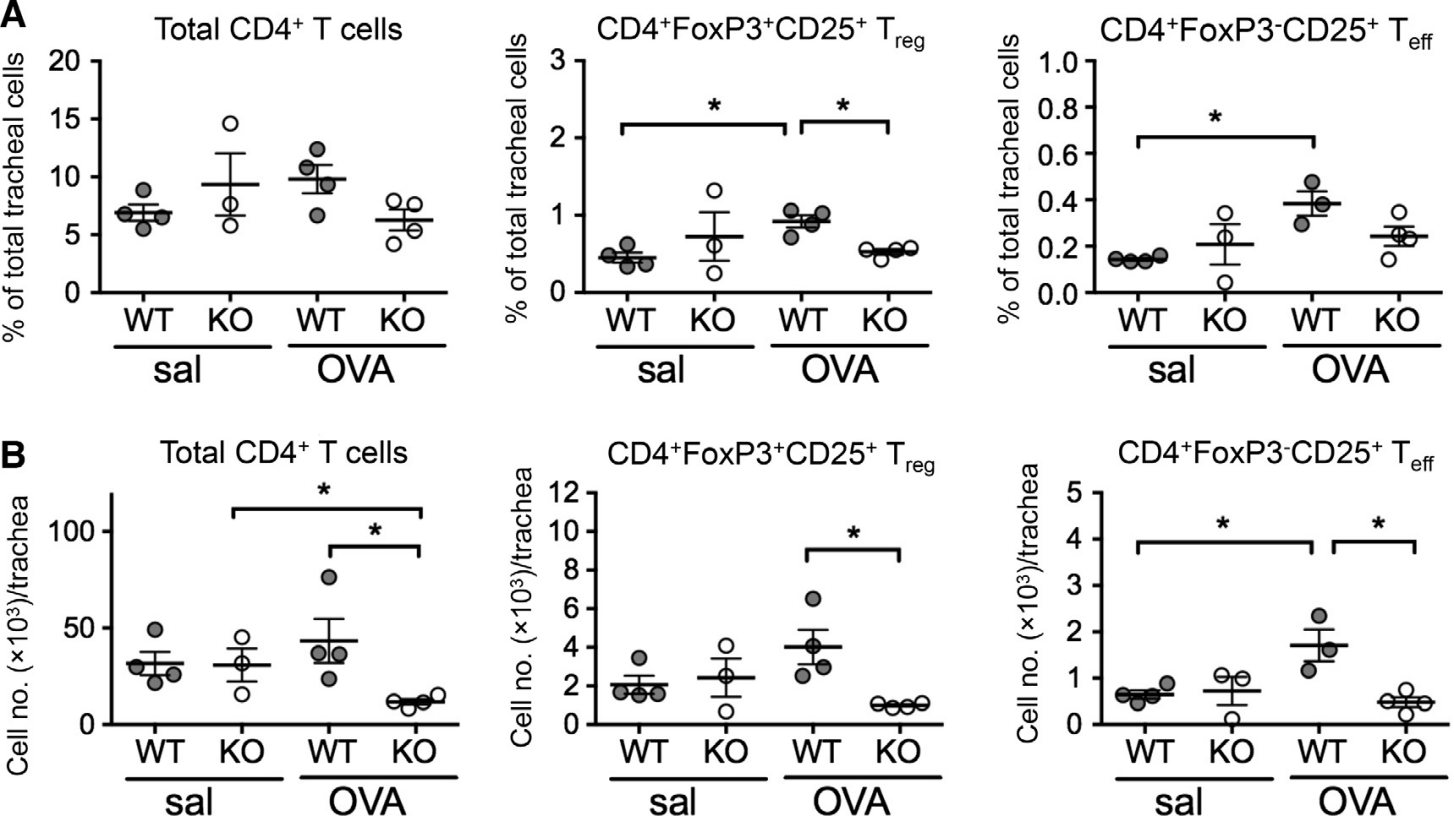
Conducting airway CD4^+^ T‐cell subset percentages and numbers in OVA‐sensitized and challenged BALB/c WT and CD103^−/−^ mice. Adult BALB/c WT and CD103 KO mice were sensitized and challenged with OVA or saline as described for Figure 1. At 24 h after final aerosol, trachea were harvested, and single cell suspensions prepared and analyzed by flow cytometry for total CD4^+^ T cells, CD4^+^ FoxP3^+^ CD25^+^ regulatory T cells (T_reg_), and CD4^+^ FoxP3^−^ CD25^+^ effector T cells (T_eff_). (A) Percentages and (B) total numbers of total, T_reg_ and T_eff_ CD4^+^ T cells in tracheas of saline or OVA‐challenged WT or CD103 KO mice. (data are a minimum of three independent experiments with *n* = 5 mice/group in pooled samples; means ^+/−^ SEM). *P <* *0.05, by one‐tailed Mann–Whitney *U*‐Test; WT, wild type; KO, knockout; OVA, ovalbumin.

Previous studies have shown an association between recruitment of CD4^+^ T cells into the airways and the development of allergic asthma in humans, and a requirement for CD4^+^ T‐cell activation and airway recruitment in the development of EAAD in BALB/c mice (Huh et al. 2003; Zosky et al. 2009). Given that CD103 is expressed on lymphocytes and that E‐cadherin is expressed on airway epithelial cells, it has been proposed that CD103 may be required for retention of pathogenic and regulatory CD4^+^ T cells within the airway epithelium (Nawijn et al. 2011). Therefore, we examined percentages and total numbers of CD4^+^ T‐cell subsets (total CD4^+^ T cells, CD4^+^ CD25^+^ FoxP3^+^ regulatory T cells (T_reg_), CD4^+^ CD25^+^ FoxP3^‐^ effector T cells (T_eff_)) in the main conducting airways of WT and CD103 KO mice by flow cytometry following OVA‐sensitization and challenge (Fig. 2). No differences in the percentages of total CD4^+^ T cells were observed in the airways of sensitized WT compared to KO mice challenged with saline or OVA (Fig. 2A, left panel). In contrast, the percentage of CD4^+^ T_reg_ cells increased in the airways of WT mice after OVA‐challenge compared to saline‐challenged WT controls (*P *<* *0.05). A similar increase in CD4^+^ T_reg_ cells was not observed in KO mice, where the percentage of T_reg_ remained significantly lower than for WT mice after OVA‐challenge (Fig. 2A, middle panel; *P *<* *0.05). Similarly, the percentages of CD4^+^ T_eff_ cells also increased in the airways of WT mice after OVA‐challenge compared to saline‐challenged WT controls (*P *<* *0.05). Again, this increase was not observed in OVA‐challenge KO mice compared to saline‐challenged KO controls (Fig. 2A, right panel).

In terms of total CD4^+^ T‐cell numbers, these were marginally but not significantly increased in the airways of OVA‐ compared to saline‐ challenged WT mice, and significantly decreased in OVA‐challenged KO mice compared to OVA‐challenged WT mice (*P *<* *0.05) and saline‐challenged KO mice (*P *<* *0.05) (Fig. 2B, left panel). Similarly, there was a nonsignificant increase in total numbers of CD4^+^ T_reg_ cells in the airways of OVA‐ compared to saline‐ challenged WT mice, and a significant decrease in OVA‐challenged KO compared to WT mice (*P *<* *0.05) (Fig. 2B, middle panel). Finally, a significant increase in the numbers of CD4^+^ T_eff_ cells was observed in the airways of OVA‐ compared to saline‐challenged WT mice (*P *<* *0.05), while there was a significant decrease in the numbers of T_eff_ cells in the airways of OVA‐challenged KO compared to WT mice (*P *<* *0.05) (Fig. 2B, right panel).

In summary, these data support a role for CD103 in the recruitment and/or retention of CD4^+^ T‐cell subsets into the airways following allergen sensitization and challenge, which correlates with a lack of AHR and BALF eosinophils in the absence of CD103 described above.

### CD103 is required for recruitment of dendritic cell subsets into the airways following aeroallergen challenge, but does not influence aeroallergen capture or DC emigration to draining lymph nodes

A central role for airway mucosal dendritic cells (AMDC) in the capture of inhaled antigens, and subsequent traffic to airway draining lymph nodes (ADLN) for surveillance by naïve CD4^+^ T cells, has been well established (Holt et al. 2008; Lambrecht and Hammad 2010). Furthermore, we and others have previously shown that subsets of AMDC, distinguished by their differential expression of CD11b and/or CD103 (CD11b^hi^ CD103^‐^ and CD11b^lo^ CD103^+^), differ in their capacities for allergen capture and trafficking to ADLN in mice (Sung et al. 2006; Beaty et al. 2007; von Garnier et al. 2007). Therefore, we reasoned that the deficiencies in CD4^+^ T‐cell recruitment to the airways described above may have been a consequence of altered AMDC numbers in the airways and/or their allergen trafficking and presentation in the ADLN.

**Figure 3:**
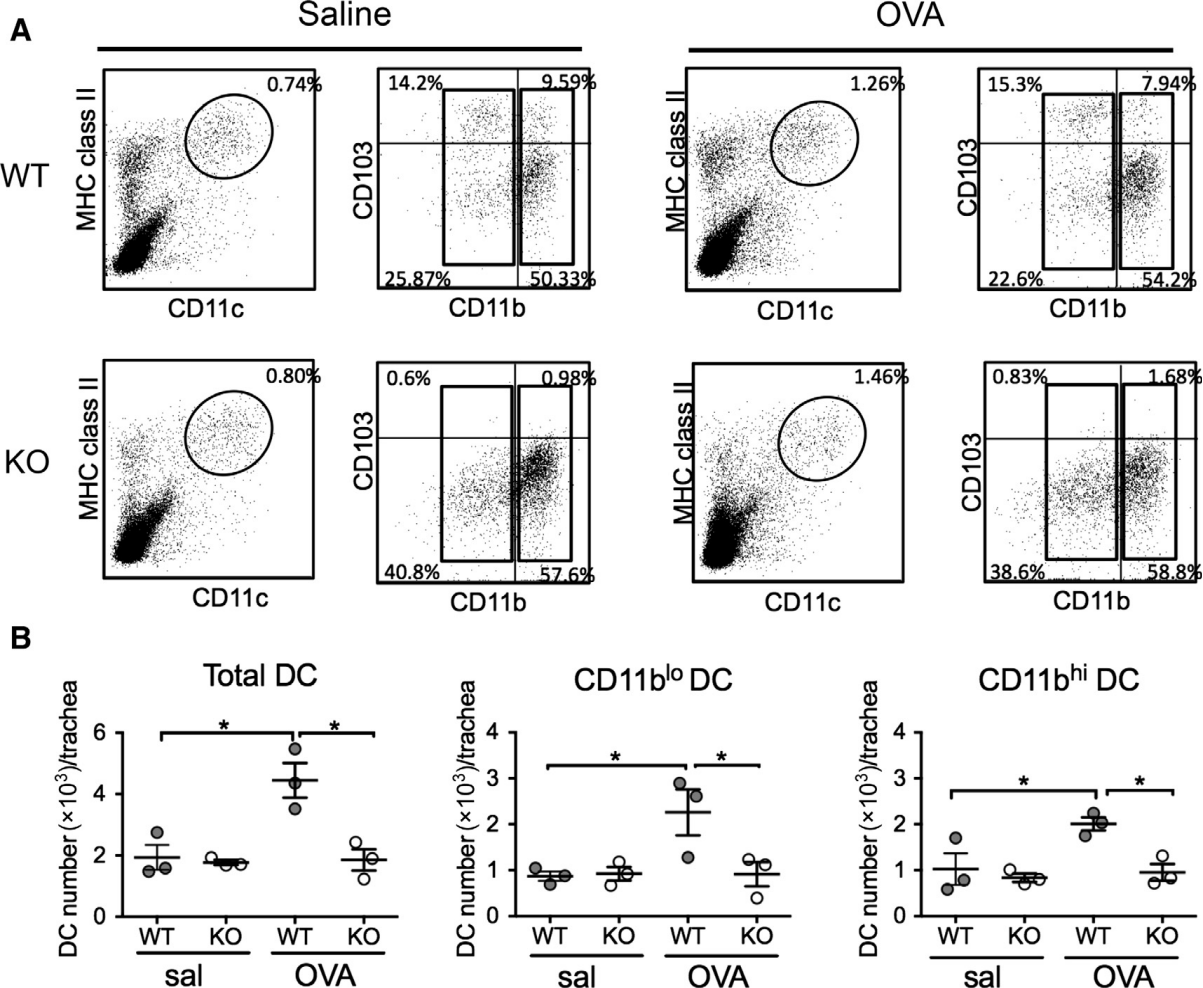
Airway dendritic cell subset phenotype and numbers in OVA‐sensitized and challenged BALB/c WT and CD103^−/−^ mice. Adult BALB/c WT and CD103 KO mice were sensitized and challenged with OVA or saline as described for Figure 1, tracheas harvested 24 h after final aerosol and single cells analyzed by flow cytometry for expression of CD11b, CD11c, MHC Class II (I/A‐E), and CD103. (A) Gating strategy for identification of airway DC populations by flow cytometry in tracheas isolated from saline‐ (left panels) and OVA‐ (right panels) challenged WT and CD103 KO mice. Airway DC were identified as I/A‐E^hi^/CD11c^hi^ after forward and side scatter gating (left panels, oval gate), then subsequently analyzed for expression of CD11b and CD103 (right panels) (representative plots of three experiments, using cells obtained from pools of 5 mice/experiment). (B) Numbers of total DC per trachea (left panel), CD11b^lo^ DC and CD11b^hi^ DC in WT and KO mice post saline‐ and OVA‐challenge. (*n* = 5 mice/group with pooled samples, for three independent experiments, data are means ^+/−^ SEM) *P <* *0.05 by one‐tailed Mann–Whitney *U*‐test. WT, wild type; KO, knockout; OVA, ovalbumin.

To address this, we first determined the percentages and numbers of total, CD11b^hi^ and CD11b^lo^ AMDC in the airways of sensitized WT and KO mice after saline‐ and OVA‐challenge (Fig. 3A). Importantly, the total percentage of CD11c^+^ MHC II^+^ AMDC was similar in WT and KO mice after both saline and OVA‐challenge, indicating that a lack of CD103 did not influence the percentages of AMDC in the airways (Fig. 3A, left panels). Likewise, despite lacking CD103, the proportions of CD11b^hi^ and CD11b^lo^ AMDC subsets in KO mice were similar to those of WT mice after both saline‐ and OVA‐challenge (Fig. 3A, right panels), confirming that a lack of CD103 did not influence total numbers or the distribution of CD11b‐expressing AMDC subsets. Next, we analyzed the total numbers of AMDC and AMDC subsets in the airways of WT and KO mice after saline‐ or OVA‐challenge (Fig. 3B). In WT mice, numbers of total AMDC showed a significant increase after OVA‐ compared to saline‐challenge (*P *<* *0.05), however, no increases in total AMDC were observed in KO mice after OVA‐challenge, the numbers of which remained significantly decreased below WT mice (*P *<* *0.05) (Fig. 3B, left panel). The changes in AMDC numbers appeared to be equally distributed across subsets of AMDC, with similar increase in both the CD11b^lo^ (Fig. 3B, middle panel; *P *<* *0.05) and CD11b^hi^ (Fig. 3, right panel; *P *<* *0.05) AMDC subsets in OVA‐ compared to saline‐challenged WT mice, whereas no increase in either AMDC subset was observed in OVA‐challenged KO mice (Fig. 3B, middle and right panels).

**Figure 4:**
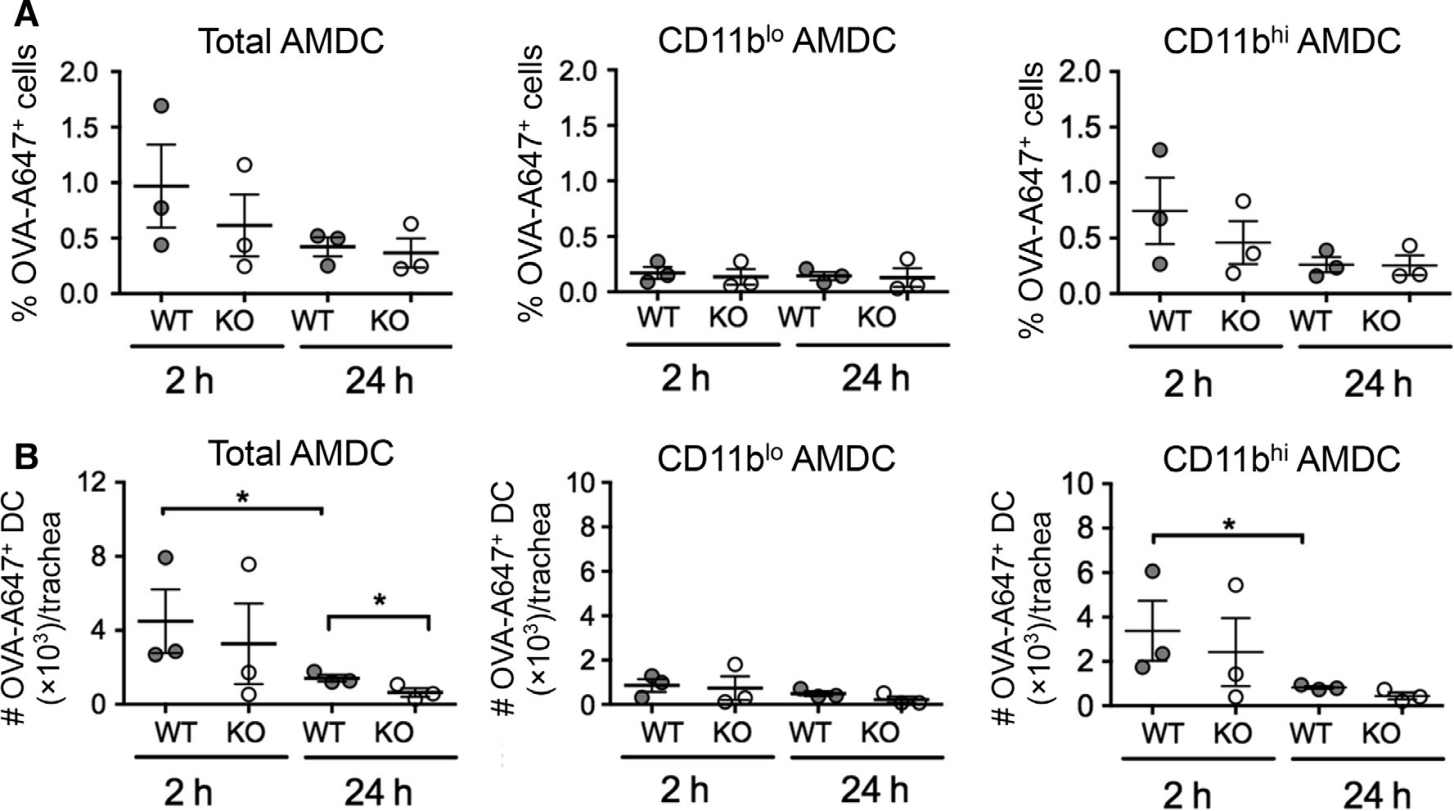
Inhaled antigen capture by airway dendritic cell subsets in BALB/c WT and CD103^−/−^ mice. Adult BALB/c WT and CD103 KO mice were sensitized with OVA in alum i.p. on days 0 and 14, and on day 21 were challenged with 50 *μ*g OVA‐A647 in 50 *μ*L saline p.n. Trachea were then harvested at 2 h and 24 h post challenge and DC analyzed by flow cytometry as described for Figure 2, along with assessment of percentages and numbers of DC containing OVA‐A647. (A) OVA uptake presented as the percentage of OVA‐A647^+^ cells among total DC, CD11b^lo^ DC, and CD11b^hi^ DC per trachea. (B) Antigen capture presented as total number of OVA‐A647^+^ total DC, CD11b^lo^ DC, and CD11b^hi^ DC per trachea. (data are from three independent experiments with *n* = 5 mice/group in pooled samples; means +/−SEM) *P <* *0.05 by one‐tailed Mann–Whitney *U*‐Test. WT, wild type; KO, knockout; OVA, ovalbumin.

Next, we examined inhaled OVA capture by WT and CD103 KO mouse AMDC, and the CD11b^lo^ and CD11b^hi^ subsets early (2 h) and late (24 h) after allergen challenge, by tracking uptake of fluorescently labeled OVA‐Alexa (A) 647 delivered i.n. into OVA‐sensitized WT and KO mice (Fig. 4). We have previously shown that, given the rapid turn‐over rates (<24 h) of AMDC (von Garnier et al. 2005), the timing for these capture experiments is important, as early time points will indicate local antigen capture by resident AMDC, while at later time points, these cells will have left the airways and migrated to ADLN (Wikstrom and Stumbles 2007). Inhaled OVA‐A647 was captured by a similar percentage of total AMDC in WT and KO mice at 2 h post challenge (Fig. 4A, left panel), with the majority of uptake by the CD11b^hi^ AMDC subset (Fig. 4A, right panel) as compared to the CD11b^lo^ AMDC subset (Fig. 4A, middle panel) in both strains of mice. In contrast, a smaller percentage of total, CD11b^lo^ and CD11b^hi^ AMDC were positive for OVA‐A647 at the 24 h time point, consistent with these cells having trafficked to the ADLN (Fig. 4A). A similar pattern was observed for total numbers of OVA‐A647^+^ AMDC subsets, with similar uptake by total (Fig. 4B, left panel) and CD11b^hi^ (Fig. 4B, right panel) AMDC of WT and KO mice at 2 h post‐OVA exposure, and little uptake by CD11b^lo^ AMDC at this time point (Fig. 4B, middle panel). Again, at 24 h, there were very few numbers of OVA‐A647^+^ AMDC remaining in the airways of either mouse strain, although numbers of OVA^+^ total AMDC of KO mice were lower than WT mice at the 24 h time point (Fig. 4B, left panel).

In summary, these data indicate that CD103 was not required for baseline homeostasis of AMDC numbers in saline‐challenged mice, whereas AMDC failed to be either recruited to, or retained within, the airways in the absence of CD103 following OVA challenge. However, similar percentages and numbers of AMDC captured inhaled OVA in both WT and KO mice at 2 h following OVA challenge, with the bulk of inhaled antigen captured by the CD11b^hi^ AMDC subset in both WT and KO mice, consistent with our previous data (von Garnier et al. 2007). Furthermore, at 24 h, the rate of egress of OVA^+^ AMDC from the airways was similar in both mouse strains, and possibly enhanced in CD103 KO mice.

### Trafficking of inhaled antigen to airway draining lymph nodes by migratory airway DC is reduced in the absence of CD103 in OVA‐sensitized and challenged mice

**Figure 5:**
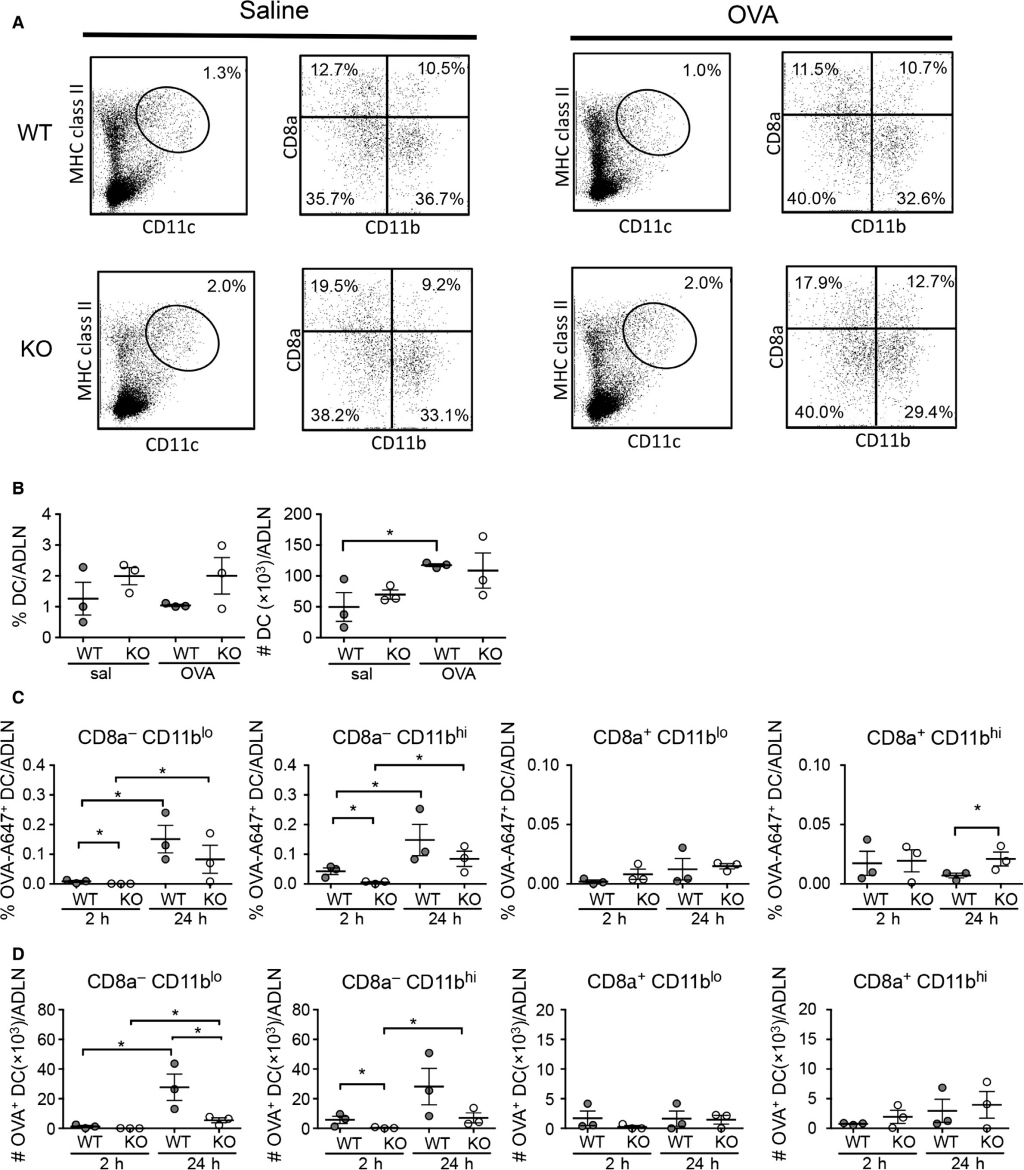
Traffic of inhaled antigen to airway draining lymph nodes in OVA‐sensitized BALB/c WT and CD103^‐/‐^ mice. Adult BALB/c WT and CD103 KO mice were sensitized with OVA in alum i.p. on days 0 and 14, and on day 21 were challenged with 50 *μ*g OVA‐A647 in 50 *μ*L saline p.n. Airway draining lymph nodes (ADLN) were then harvested at 2 h and 24 h post challenge and DC analyzed by flow cytometry as described for Figure 2, including labeling for CD8*α* ^‐^ migratory and CD8*α* ^+^ resident DC, along with assessment of percentages and numbers of DC containing OVA‐A647. (A) Gating strategy for identification of ADLN DC populations by FACS in saline and OVA‐challenged WT and CD103 KO mice. DC were identified as I–A/E^hi^ CD11c^hi^ cells (left panels), and then subsequently gated for expression of CD8*α* and CD11b (right panels). (B) Total DC percentages and numbers per ADLN in WT and CD103 KO mice post saline‐ and OVA‐challenge. (C) Percentages of CD8*α* ^‐^CD11b^lo^, CD8*α* ^‐^CD11b^hi^, CD8*α* ^+^CD11b^lo^, CD8*α* ^+^CD11b^hi^ ADLN DC subpopulations positive for OVA‐Alexa 647, presented as mean percentage frequency +/− SEM of total cells in ADLN (*n* = 5 mice/group with pooled samples, for three independent experiments) (D) Total numbers of CD8*α* ^‐^CD11b^lo^, CD8*α* ^‐^CD11b^hi^, CD8*α* ^+^CD11b^lo^, CD8*α* ^+^CD11b^hi^ ADLN DC subpopulations positive for OVA‐Alexa 647, presented as mean total number positive cells +/− SEM per ADLN (*n* = 5 mice/group with pooled samples, for three independent experiments). *P <* *0.05 by one‐tailed Mann–Whitney U‐Test. WT, wild type; KO, knockout; OVA, ovalbumin.

Delivery of inhaled antigens from the airways to the ADLN by antigen‐loaded migratory DC is required for activation of naive antigen‐specific T cells and their homing to the airway mucosa to induce inflammation (Holt et al. 2008; Lambrecht and Hammad 2010). In lymph nodes, DC can be divided into CD8*α* ^−^ migratory DC and CD8*α* ^+^ resident DC, the former being the primary driver of CD4^+^ T‐cell responses via classical MHC Class II restricted presentation pathways, while the latter act in part to cross‐present soluble antigens to CD8^+^ T cells (Miller et al. 2012). Therefore, we next examined the traffic of inhaled antigen to ADLN, and its distribution among migratory (CD8*α* ^−^) and resident (CD8*α* ^+^), CD11b^hi^ or CD11b^lo^ ADLN DC subsets, by i.n. exposure of OVA‐sensitized wild‐type and KO mice to fluorescently labeled OVA‐Alexa 647 and determining the distribution of OVA among ADLN DC subsets 2 h and 24 h later (Fig. 5A). Total ADLN cell numbers at 24 h post OVA‐challenge were significantly increased in the WT OVA‐challenged compared to the WT saline challenged (11.3 × 10^6^ cells/ADLN and 3.8 × 10^6^ cells/ADLN, respectively; *P *<* *0.001), no significant increases were observed for KO mice (data not shown). While the percentages of DC in the ADLN did not change significantly between saline‐ and OVA‐challenged WT or KO mice (Fig. 5A and B, left panel), the total DC number per lymph node was significantly increased in OVA‐challenged compared to saline‐challenged WT mice (*P *<* *0.05) (Fig. 5B, right panel). Total DC numbers were also elevated in OVA‐challenged KO mice compared to saline‐challenged controls, however, this was not statistically significant (Fig. 5B, right panel). In addition, there were no significant differences in the absolute percentages of CD8*α* ^−^ CD11b^lo^ and CD8*α* ^‐^ CD11b^hi^ or CD8*α* ^+^ CD11b^hi^ and CD8*α* ^+^ CD11b^lo^ ADLN DC between OVA‐challenged WT and KO mice at 2 h and 24 h post‐OVA challenge (Fig. 5A).

Next, we determined the percentage of each DC population positive for OVA‐A647 at 2 h and 24 h after i.n. delivery into OVA‐sensitized mice (Figs. 5C and 5D). For both WT and KO mice, the percentages of OVA‐A647^+^ CD8*α* ^‐^ CD11b^lo^ and CD8*α* ^‐^ CD11b^hi^ migratory ADLN DC significantly increased at 24 h compared to 2 h post‐OVA challenge, although the percentage OVA‐A647^+^ DC were lower in KO compared to WT mice at the 24 h time point. (Fig. 5C, left panels; *P *<* *0.05). Interestingly, although at low levels, the percentages of OVA‐A647^+^ CD8*α* ^−^ CD11b^lo^ and CD8*α* ^‐^ CD11b^hi^ ADLN DC at the 2 h time point were significantly elevated in WT compared to the KO mice (Fig. 5C, left panels; *P *<* *0.05). No significant changes in the percentages of OVA‐A647^+^ CD8*α* ^+^ CD11b^lo^ resident ADLN DC were observed at 2 h or 24 h post challenge, however, there was a minor although significant increase in the percentage of OVA‐A647^+^ CD8*α* ^+^ CD11b^hi^ resident ADLN DC in KO compared to WT mice at 24 h post challenge (Fig. 5C, right panels). A similar trend was observed for total numbers of OVA‐A647^+^ ADLN DC (Fig. 5D), although in this case, the total numbers of OVA‐A647^+^ CD8*α* ^−^ CD11b^lo^ migratory ADLN DC were significantly lower in KO compared to WT mice at the 24 h time point (Fig. 5D, left panel; *P *<* *0.05).

In summary, these data indicate that in WT mice, inhaled OVA is carried to ADLN by both the CD11b^lo^ and CD11b^hi^ migratory CD8*α* ^‐^ ADLN DC subsets within 24 h after OVA exposure, with some OVA appearing as early as 2 h, consistent with our previous findings (Wikstrom et al. 2006). In contrast, both the percentage and total numbers of OVA^+^ migratory CD8*α* ^‐^ ADLN DC were reduced in OVA‐sensitized and challenged KO mice, both for the CD11b^lo^ and CD11b^hi^ subsets at 2 h and 24 h post‐OVA exposure.

### CD4^+^ T‐cell subset responses are normal or enhanced in airway draining lymph nodes of sensitized and challenged mice in the absence of CD103

**Figure 6:**
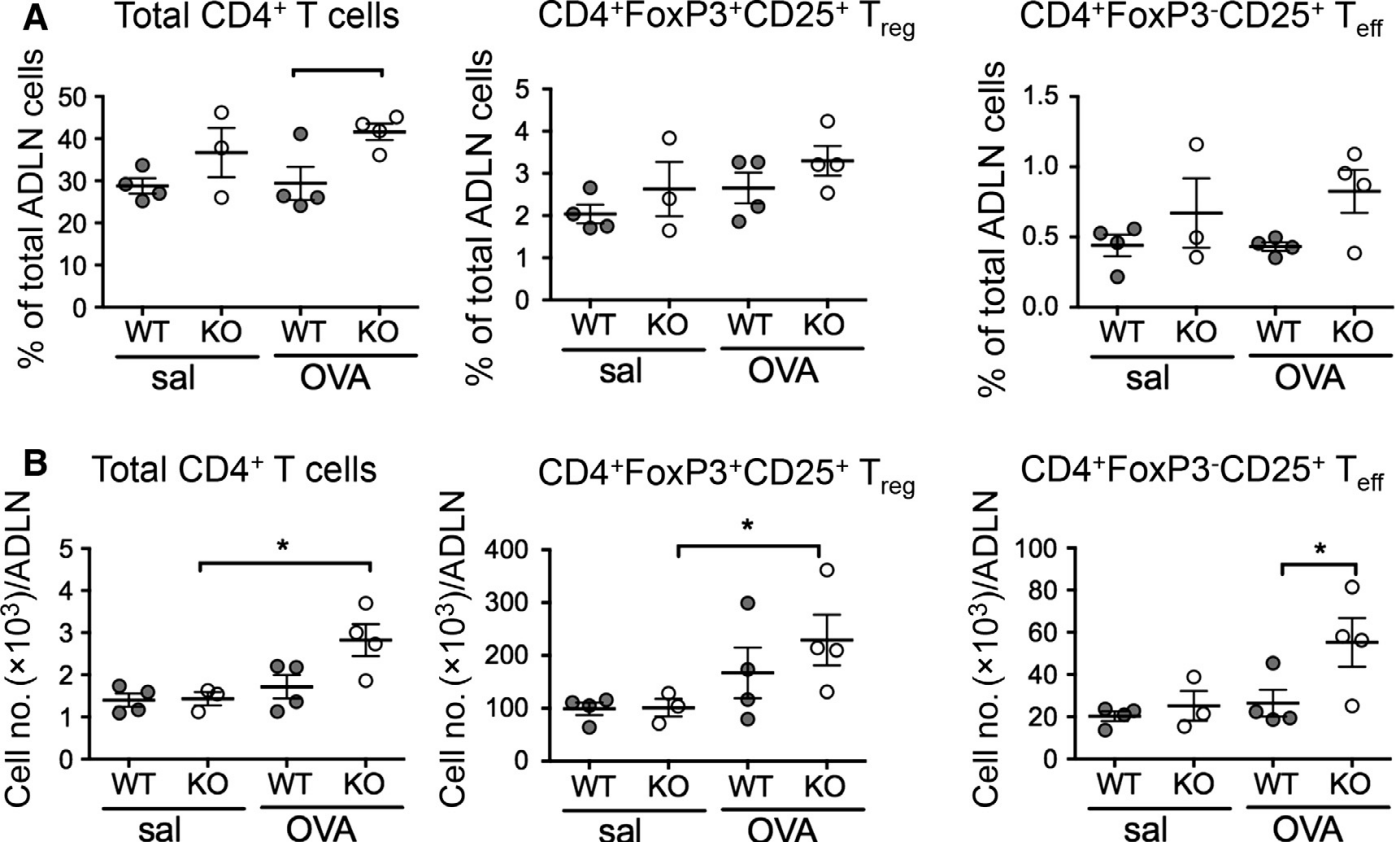
Airway draining lymph node CD4^+^ T‐cell subset percentages and numbers in OVA‐sensitized and challenged BALB/c WT and CD103^−/−^ mice. Adult BALB/c WT and CD103 KO mice were sensitized and challenged with OVA or saline as described for Figure 1. At 24 h after final aerosol, ADLN were harvested, and single cell suspensions prepared and analyzed by flow cytometry for total CD4^+^ T cells, CD4^+^ FoxP3^+^ CD25^+^ T_reg_ and CD4^+^ FoxP3^‐^ CD25^+^ T_eff_. (A) Percentages and (B) total numbers of total, T_reg_ and T_eff_ CD4^+^ T cells in ADLN of saline‐ or OVA‐challenged WT or CD103 KO mice. (data are three independent experiments with *n* = 5 mice/group in pooled samples; means +/− SEM). *P <* *0.05 by one‐tailed Mann–Whitney *U*‐Test. ADLN, airway draining lymph node; WT, wild type; KO, knockout; OVA, ovalbumin.

The OVA trafficking data described above suggested that altered migration of antigen‐bearing airway DC to the ADLN in KO mice may have an impact on CD4^+^ T‐cell priming and the generation of activated CD4^+^ effector T‐cell subsets. Therefore, we next examined the percentages and total numbers of total CD4^+^ T cells, CD25^+^ FoxP3^+^ regulatory CD4^+^ T cells (T_reg_), and CD25^+^ FoxP3^‐^ effector CD4^+^ T cells (T_eff_) in the airway draining lymph nodes (ADLN) of OVA‐sensitized KO and WT mice following OVA‐challenge (Fig. 6). There were no changes in the percentages of total CD4^+^ T cells, CD4^+^ CD25^+^ FoxP3^+^ T_reg_, or CD4^+^ CD25^+^ FoxP3^‐^ T_eff_ in response to OVA‐challenge in either WT or KO mice compared to their saline‐challenged controls (Fig. 6A). However, there was a significantly increased percentage of total CD4^+^ T cells in OVA‐challenged KO mice compared to OVA‐challenged WT mice (*P *<* *0.05) (Fig. 6A, left panel). For total cell numbers, OVA‐challenged WT mice showed no differences to saline‐challenged WT control mice for any of the CD4^+^ T‐cell subsets (Fig. 6B). However, there were significantly increased numbers of total CD4^+^ T cells (Fig. 6B, left panel) and CD4^+^ CD25^+^ FOXP3^+^ T_reg_ cells (Fig. 6B, middle panel) in the OVA‐challenged KO mice compared to saline‐challenged KO control mice. Furthermore, there were elevated numbers CD4^+^ FoxP3^‐^ CD25^+^ T_eff_ cells in the OVA‐challenged KO mice in comparison to OVA‐challenged WT mice. (Fig. 6B, right panel).

In summary, the numbers of CD4^+^ T‐cell subsets in the ADLN of sensitized and OVA‐challenged KO mice were increased when compared to WT mice, suggesting that a lack of CD103 did not restrict CD4^+^ T‐cell subset activation or expansion in the ADLN in response to inhaled allergen, and may perhaps have lead to their increased retention or reduced emigration.

## Discussion

In this study, we have examined the role of CD103 in the pathogenesis of allergic airways disease using an ovalbumin‐induced mouse model of EAAD in BALB/c CD103^−/−^ (KO) mice. We found a lack of CD103 lead to significantly reduced clinical symptoms of EAAD, including reduced AHR and airway eosinophilia, when compared to wild‐type (WT) BALB/c mice, suggesting a pathogenic role for CD103 in the pathogenesis of allergic asthma in this model. Our data appear to be contradictory to those of Bernatchez et al. (2015) who showed an exacerbation of the clinical symptoms of OVA‐induced EAAD in CD103 KO mouse model. The reasons why the two models differ remains unclear, however, there were a number of differences in experimental design that may be informative: Firstly, the Bernatchez study was performed in CD103 KO mice on a C57BL/6 (B6) background rather than the BALB/c background used in this study. Genetic background is known to have a major impact on EAAD expression in mice, with B6 mice generally showing attenuated AHR compared to BLAB/c mice (Ewart et al. 2000; Elena et al. 2003). Secondly, the number of antigen challenge doses is known to significantly affect the severity of methacholine responses in the OVA model: the Bernatchez study used a total of five OVA aerosol challenges, whereas we used a single OVA challenge (Zosky et al. 2004). This was reflected by the high percentages of eosinophils in BALF observed in the Bernatchez study (maximal of 70%) compared to ours (maximal of 40%), which may have impacted on the overall expression of clinical symptoms. Finally, sexual dimorphism is known to play a significant role in the expression of respiratory inflammatory diseases in mice, with female mice developing more severe disease in the OVA‐induced allergic airways inflammation model (Melgert et al. 2005). While the Bernatchez study specified that sex‐matched mice were used, they did not specify which sex was used for their OVA studies. While we used only female mice in this study, it is possible that sexual dimorphism may have had an impact on the inflammatory responses between the two studies.

Interestingly, we found that CD103 KO mice developed significantly elevated levels of circulating OVA‐specific IgE following OVA sensitization when compared to WT BALB/c mice, suggesting that the reduction in clinical symptoms seen in CD103 KO mice were not simply due to a deficiency in systemic sensitization to OVA, or priming of allergen‐specific Th2 cells required for anti‐OVA IgE class switching. The reason for the elevated IgE response in CD103 KO mice is unknown, but may suggest a role for CD103 in regulating Th2 priming or CD4^+^ T‐cell recirculation to germinal centers. While we did not perform lavage cytokine analysis in this study, our data showing that systemic IgE priming to OVA was intact or exacerbated in CD103 KO mice in the face of reduced local airway inflammation and AHR suggests a role for CD103 in regulating the clinical features of EAAD independently of systemic allergen sensitization and IgE. While we were unable to provide a mechanistic basis for this in the current study, previous studies have shown that AHR and inflammation are not strictly correlated in human allergic asthmatic patients (Crimi et al. 1998), and that inflammation and AHR may be differentially regulated, with the traits controlled at different genetic loci, in mouse models of EAAD (Brewer et al. 1999; Ewart et al. 2000; Poynter et al. 2004). Our data extend these findings, suggesting that CD103 plays a role in regulating local airway inflammation and AHR independently of systemic IgE production.

CD103 can be expressed on both CD4 T cells and DC, two cell types that are centrally implicated in the pathogenesis of allergic airways disease (Holt et al. 2008). Furthermore, E‐cadherin, the ligand for CD103, is expressed on airway epithelial cells and appears to be important in the retention and localization of both CD4^+^ T cells and DC within the epithelium at the site of antigen exposure (Nawijn et al. 2011). For these reasons, we hypothesized that CD103 may play a role regulating local airway DC or CD4^+^ T‐cell function or activation, or migration of DC and subsequent CD4^+^ T‐cell priming to inhaled antigens in airway draining lymph nodes. Our data show that the numbers of CD4^+^ T cells in the airways of sensitized and challenged CD103 KO mice, including effector and regulatory T cells, were significantly reduced when compared to wild‐type mice, suggesting that either these T cells failed to be recruited to the airways following challenge, or failed to be retained with the airway mucosa. Previous studies have shown a requirement for CD103 for retention of CD4^+^ (regulatory) T cells in the skin during bacterial‐induced inflammation (Schön et al. 1999), while other studies have shown an essential role for CD4^+^ T‐cell function in mediating the major clinical signs of EAAD during the effector phase of the disease (Li et al. 1999). Our data are consistent with these studies, suggesting that CD103 is required for recruitment and/or retention of effector CD4^+^ T‐cell subsets within the airways that are required to mediate the local expression of clinical disease, including AHR and recruitment of eosinophils.

In addition to expression by T cells, CD103 is also expressed on subsets of DC, and in particular those proposed to reside within the airway epithelium (Hammad and Lambrecht 2008). Interactions between DC and T cells are crucial to the initiation and maintenance of inflammation to inhaled allergens (Huh et al. 2003), with two major subsets of CD11b^hi^ CD103^‐^ and CD11b^lo^ CD103^+^ respiratory DC have been described, each with proposed differing roles in regulating airway inflammation (Sung et al. 2006; Beaty et al. 2007). Initially, we addressed the issue of whether the balance of airway DC subsets was disrupted in the absence of CD103 as a potential explanation for the disrupted CD4^+^ T‐cell responses we observed in CD103 KO mice. Importantly, in sensitized but saline‐exposed mice, total numbers of airway DC and the proportions of CD11b^lo^ and CD11b^lo^ airway DC subsets were similar between KO and WT mice. However, following specific allergen challenge, CD103 KO mice failed to recruit DC subsets into the airways to the same extent as WT mice, with DC numbers not increasing above steady‐state levels. As recruitment of DC to the airways is a hallmark feature of airway mucosal inflammatory responses (Stumbles et al. 2001), our data indicate that CD103 is critical for recruitment of DC to the airways during inflammation, however, is not required for baseline homeostasis of airway DC numbers or subset balance. Consistent with this, inhaled allergen capture by CD11b^lo^ and CD11b^hi^ DC subsets in the airways was not markedly disrupted in CD103 KO mice at early time points (2 h) after exposure, and we also observed similar rates of egress of antigen‐positive cells from the airways at later time points (24 h) after exposure, although with some suggestion that egress was more rapid in CD103 KO mice.

However, despite apparently normal levels of allergen capture by DC in the airways of CD103 KO mice, we did observe a significant decrease in the absolute numbers of allergen‐laden migratory (CD8*α* ^‐^) DC reaching the airway draining lymph nodes of CD103 KO mice compared to WT mice 24 h after allergen challenge. Nevertheless, this decrease was not reflected by a decreased expansion of effector or regulatory CD4^+^ T‐cell subsets in the draining lymph nodes, with CD103 KO mice showing an equivalent or enhanced increase in numbers of total, regulatory and effector CD4^+^ T cells after allergen exposure when compared to WT mice. This suggests that sufficient antigen must have reached the lymph nodes of CD103 KO mice in order to drive CD4^+^ T‐cell activation and expansion, and indeed we did observe elevated percentage frequencies of antigen‐laden CD8*α* ^‐^ DC in the ADLN of KO mice at 24 h compared to 2 h post‐i.n. delivery, but at lower levels than in WT mice. In addition, an absence of CD103 on T cells may lead to a retention of activated T cells in the draining lymph nodes, or may disrupt CD103‐mediated regulatory pathways that have previously been described involving induction of beta‐catenin in CD103^+^ DC (Jiang et al. 2007; Manicassamy et al. 2010).

In summary, we have shown that CD103 plays an important role in the pathogenesis of experimental allergic airways disease in pro‐allergic BALB/c mice through regulation of airway expression of AHR and eosinophilia, but not in systemic sensitization to allergen or induction of allergen‐specific IgE. We show that CD103 was required for the recruitment and/or expansion of CD4^+^ T cells and subsets of DC within the airways during inflammation, but was not required for maintenance of airway DC subset homeostasis in the steady‐state and its absence did not influence the capacity of airway DC to capture inhaled allergen. An absence of CD103 did, however, reduce the numbers of antigen‐laden DC reaching draining lymph nodes, although this was not reflected by a reduced expansion of CD4^+^ T cells with lymph nodes. Speculatively, our data suggest a dual role for CD103 in recruiting DC to airways and guiding their migration to draining lymph nodes during allergic inflammation, as well for guiding activated CD4^+^ T cells from lymph nodes back to the airways in order to mediate the local expression of allergic airways disease.

## Conflict of Interest

The authors declare no conflicts of interest.
